# Differentiation of Gram-Negative Bacterial Aerosol Exposure Using Detected Markers in Bronchial-Alveolar Lavage Fluid

**DOI:** 10.1371/journal.pone.0007047

**Published:** 2009-09-16

**Authors:** David Wunschel, Bobbie-Jo Webb-Robertson, Charles W. Frevert, Shawn Skerrett, Nat Beagley, Alan Willse, Heather Colburn, Kathryn Antolick

**Affiliations:** 1 Chemical and Biological Signature Sciences, Pacific Northwest National Laboratory, Richland, Washington, United States of America; 2 Computational Biology & Bioinformatics, Pacific Northwest National Laboratory, Richland, Washington, United States of America; 3 Division of Pulmonary and Critical Care Medicine, University of Washington School of Medicine, Seattle, Washington, United States of America; 4 Department of Comparative Medicine, University of Washington School of Medicine, Seattle, Washington, United States of America; 5 Computational Mathematics, Pacific Northwest National Laboratory, Richland, Washington, United States of America; Johns Hopkins School of Medicine, United States of America

## Abstract

The identification of biosignatures of aerosol exposure to pathogens has the potential to provide useful diagnostic information. In particular, markers of exposure to different types of respiratory pathogens may yield diverse sets of markers that can be used to differentiate exposure. We examine a mouse model of aerosol exposure to known Gram negative bacterial pathogens, *Francisella tularensis novicida* and *Pseudomonas aeruginosa*. Mice were subjected to either a pathogen or control exposure and bronchial alveolar lavage fluid (BALF) was collected at four and twenty four hours post exposure. Small protein and peptide markers within the BALF were detected by matrix assisted laser desorption/ionization (MALDI) mass spectrometry (MS) and analyzed using both exploratory and predictive data analysis methods; principle component analysis and degree of association. The markers detected were successfully used to accurately identify the four hour exposed samples from the control samples. This report demonstrates the potential for small protein and peptide marker profiles to identify aerosol exposure in a short post-exposure time frame.

## Introduction

Developing molecular markers of infectious disease is a subject of intense interest. Early detection of infection, prior to the onset of symptoms and pathology is the ultimate goal. The time scale for early detection is a few hours to days after infection before common symptoms of illness (e.g. fever, malaise, and congestion) appear [1]. This time scale also precedes the mounting of an effective acquired immune response and is of particular interest for rapid detection of exposure. Aerosol exposure to infectious agents is a commonly discussed bioterrorism scenario because of the potential for affecting large numbers of people [1], [2], [3], [4]. Many of the diseases, such as anthrax, also require treatment within the first 24 hours, prior to the onset of symptoms, to be most effective [5]. A number of bacterial agents are considered as potentially being used in bioterror, including *Francisella tularensis*, the causative agent of tularemia [6]. In this report, we focus on the tools to examine the early markers of host response to aerosol exposure in a mouse model of tularemia.

The early events in the infection and immune response typically involve interaction by the pathogen with epithelial layers and resident professional phagocytic cells [7]of the skin, gastrointestinal or respiratory tracts. The result is often activation of these cell types to produce antimicrobial compounds and inflammatory mediators. The protein/peptide markers immediately expressed and secreted after infection play important roles during the innate immune response [8], [9], [10]. A variety of cytokines, chemokines and defensins have been postulated to serve as important markers of response for diagnostic purposes [8], [10], [11]. The technology for profiling cytokines and chemokine markers has generally employed affinity reagents because these markers are often present at picomolar concentrations [12], [13]. The challenge of detecting cytokines becomes larger when examining small or dilute samples, such as breath condensate [13], [14]. A further complication is often finding the affinity reagents to measure a range of markers, especially in certain host species [15]. Furthermore, the use of affinity reagents places limits on marker discovery to only those proteins with the corresponding antibody reagents used in the assay.

The pathogenesis of bacterial disease in the lungs can involve both host and pathogen proteases [16], [17], [18], [19], [20]. Host proteases are required as an activation event for cytokines and defensins [19], [20], [21]. However bacterial proteases have also evolved to inactivate or modify important immune effectors such as defensins and immunoglobulin [18], [22], [23]. Bacterial proteases are also directly involved in tissue damage [16], [17]. The combination of expressed immune peptides as well as protease derived degradation products can potentially generate a number of signature peptides in response to infection. These peptides may serve as effective discriminators of host-bacteria interaction and were targeted for analysis.

Biomarker discovery and profiling by direct matrix assisted laser desorption/ionization (MALDI) mass spectrometry (MS) or a more recent variation, surface enhanced laser desorption/ionization (SELDI) MS has gained wider application for comparison of clinical samples. Several applications have been reported including profiling body fluids for detection of cancer and infectious disease related patterns [24], [25]. One area of broad application for MALDI-MS has been in bacterial identification [26], [27], [28], [29]. Mass lists of easily extracted and detected markers from cultured bacterial samples form the basis for comparison and ultimately, identification of organisms. This requires construction and searching of large marker databases [30], [31]. Two commercial systems have been designed for this purpose by Bruker Daltonics and Waters-Micromass [32], [33].

Among the strengths of direct MALDI analysis is small sample size, relative speed of analysis, ease of replication with an inherent ability to multiplex the analysis. Furthermore, superior mass resolution and accuracy can be achieved when compared to gel based separations, as well as an increased sensitivity for low mass markers [34], [35]. However the trade-offs are that MALDI and SELDI have a much more limited mass range and analyte suppression is seen for low abundance ions. Different matrices also provide differential ionization efficiency for individual proteins and peptides. To address the problem of suppression, subsets of markers are captured on surfaces or magnetic beads based on general chemical properties such as hydrophobicity or ion exchange. Performing the cleanup off the SELDI target allows flexibility in the amount of material applied and the MS platform used for analysis.

Building on this approach we investigate combination of an acid extraction and two stage solid phase sample extraction coupled to a MALDI-MS analysis to profile markers of response to infection in the lung. The targeted capture of biomarkers used in this study is based on known characteristics of antimicrobial peptides, principally their acid solubility and cationic nature. In this report, we investigate the use of an acid extraction in conjunction with a two step solid phase extraction (SPE) using strong cation exchange and reverse phase to profile markers using MALDI-MS. This approach is applied to investigating patterns of markers produced in response to pathogens.

A mouse model of aerosol exposure and infection for three organisms was examined using this technique. Two pathogens *Francisella tularensis* ssp *novicida (F. novicida)*, and *Pseudomonas aeruginosa* were used along with a non-pathogenic mutant of *F. novicida* that lacks the transcriptional regulator mglA. *F. novicida* infection in mice serves as a model for human infection with the category A pathogen, *F.tularensis* ssp *tularensis* [36]. *F. novicida* is highly virulent in mice with any exposure, while very rarely pathogenic in man [37].

The major endotoxin component of the gram negative cell, the lipid A portion of the lipopolysaccharide (LPS), elicits a poor host cytokine response for *Francisella* pathogens [36], [38]. This is thought to limit the innate immune response to this intracellular pathogen. Portions of the pilis IV regulatory apparatus were also found to be essential for pathogenesis of this organism [39]. The deletion of the transcriptional regulator for this apparatus creates a non-pathogenic mutant in mice and is of interest in studying the host response. While other pathogenesis factors are likely to exist, these factors aid in the evasion of the immune response to *Francisella*.

The goal of this study was to determine if distinct profile of exposure markers could be detected using the capture and purification of peptide markers prior to MALDI-MS analysis. The study was conducted using an experimental design to limit effects of exposure time or order prior to sample collection. Four mice were studied for each treatment and time group with replication and randomization of the MALDI-MS data collection. Following data collection, tools for spectral alignment and peak picking were used for data reduction. The experimental design provided an effective tool to allow discovery of significant markers using a non-parametric analysis of variance.

## Results

### Bronchial Alveolar Lavage (BAL) Fluid Cell Measurements

The bacterial cell load was assessed through direct culture in separate experiments. As seen in table 1, the number of colony forming units was measured to be one to four thousand for both the wild-type (Fn) and attenuated (Fn-ATT) *F. novicida* with significantly larger dose measured for the *P. aeruginosa* (Pa) exposure.

**Table 1:** Measurement of bacterial deposition in colony forming units (CFU) per lung for virulent (Fn) and attenuated (Fn-ATT) *F. novicida* and *P. pseudomonas* (Pa) with standard error of the mean (SEM) indicated for each measurement.

	Fn-1	Fn-2	Fn-ATT-1	Fn-ATT-2	Pa
CFU/lung	4248	3030	3827	1262	126127
SEM	1215	214	815	408	14239

**Figure 1:**
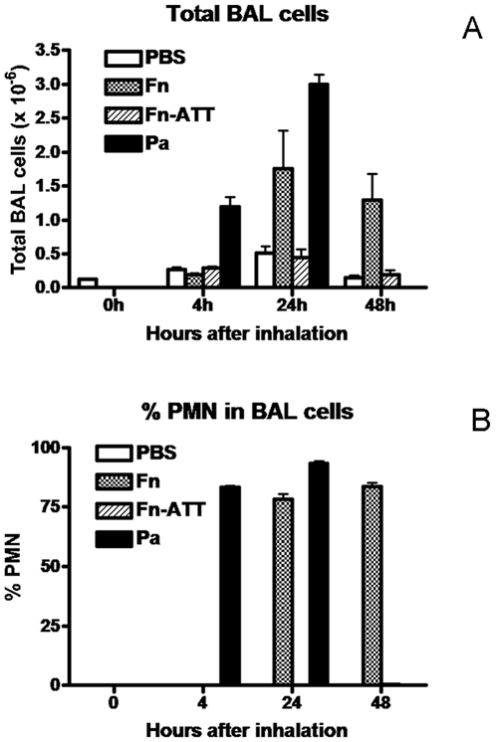
Total number of mononuclear (MN) and poly morphonuclear (PMN) cells counted in the BAL fluid at 4 h, 24 h, and 48 h for Fn and Fn-ATT and at 4 h and 24 h for Pa (A). Error bars indicate the standard error of the mean (SEM) for each. Percent PMN cells in BAL fluid (B).

The response to bacterial exposure was measured by the number of immune cells found in the BAL fluid for four mice in each group. Both mononuclear (MN) and poly morphonuclear (PMN) cells were counted in the BAL at 4 h, 24 h, and 48 h for Fn and Fn-att and at 4 h and 24 h for Pa. (Figure 1). The total cell counts revealed differences between each type of exposure. At 4 hours, only the Pa exposure showed a significant increase in cell count while at 24 hours both Pa and Fn showed a significant increase in immune cell recruitment. Much of this data can be explained by differences in PMN count alone (Figure 1B). No PMN are seen in the 0 hour control and only the Pa sample contained PMN at 4 hrs. The Pa and Fn samples showed PMN at 24 hrs similar to the total cell count data.

### MALDI-MS Mass Measurement

Mass spectral comparisons rely on reproducible measurements and assessment of the variability of those measurements. Mass calibration for the plate was done using plate locations to calibrate an area of the plate with each sample being within three plate locations of the calibrant. Sinapinic acid was also chosen as the MALDI matrix because of the increased instrument performance with greater peak resolution over another common matrix, α-hydroxy cinnamic acid (data not shown). Typical peak resolutions were estimated using external calibrant peaks appearing in the same *m/z* range as many of the markers observed, such as ubiquitin ([M+H] ^+^ *theo. m/z* 8565.89) and cytochrome c ([M+2H] ^+^ *m/z* 6181.05). Mass resolution of 500 +/− 50 was observed as well as an average mass measurement accuracy of 25 ppm and precision of 130 ppm. The peak statistics for sample markers indicated slightly lower performance for a commonly observed marker in BALF, *m/z* 3349.4. The standard deviation of the mass measurement was 0.92 *m/z* (precision of 274 ppm) was determined over fifteen replicate mass measurements with an average peak resolution of 390 +/− 30.

To increase consistency of data acquisition, ground stainless steel sample plates were used to provide more reproducible crystal formation with the chosen matrix. However, despite the mass resolution advantages, sinapinic acid tends to form less homogeneous crystals than α-hydroxy cinnamic acid. Therefore it was determined that 500 laser shots needed to be acquired in 50 shot intervals to get a representative mass spectrum for each spot.

### Feature Extraction

**Figure 2:**
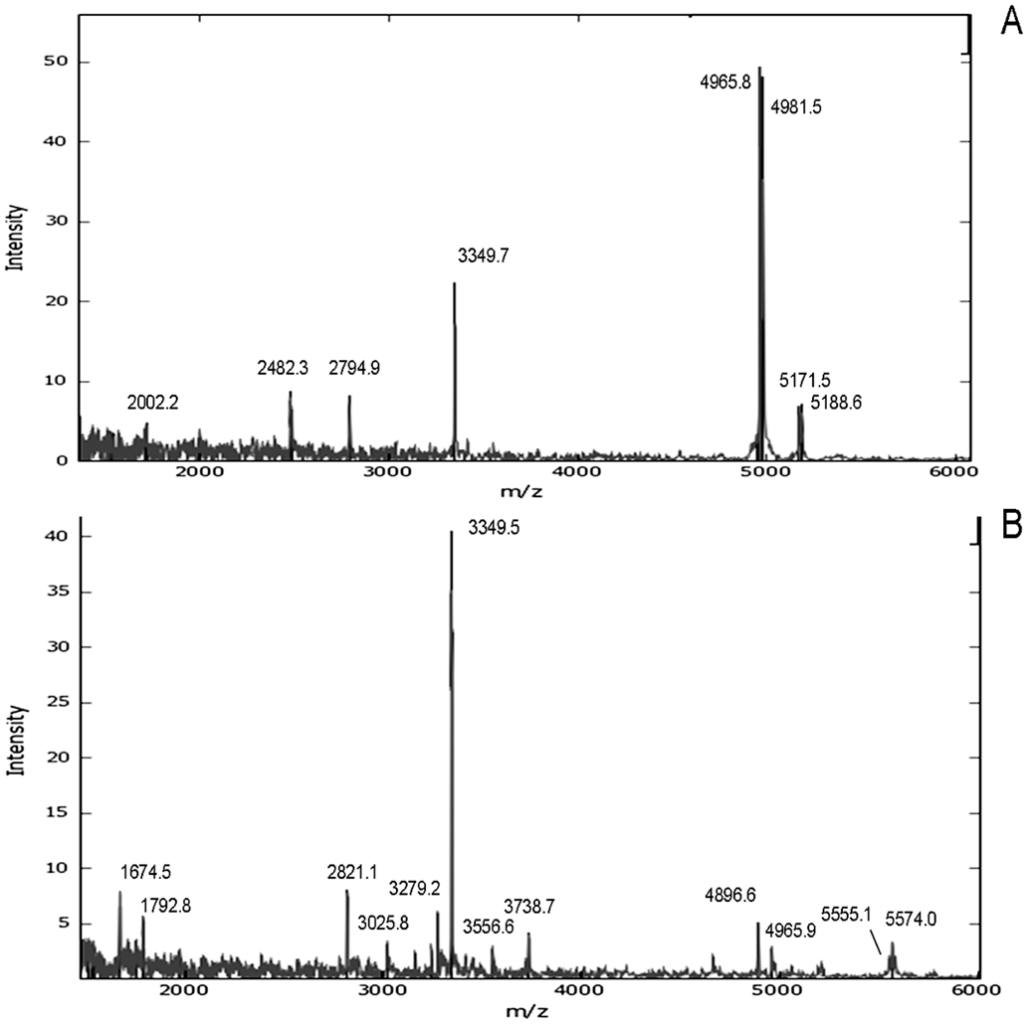
Example mass spectra comparing of a portion of the mass range for a Fn-4 hrs sample (A) and a Pa-4 hrs sample (B).

Following acquisition, the data were analyzed based on the set of 219 binary peaks. Peaks were picked using an approach previously applied to MALDI-MS spectra of bacterial samples [40]. For this analysis, all control time points were combined as a single group totaling 12 mice. Of the 219 peaks, only ∼5% were deemed significant by Kruskal-Wallis with a Bonferroni correction for multiply hypothesis tests, Table 2. Examples of MALDI-MS data used in the study are provided in Fig. 2 showing comparison of Fn-4 hr sample to a Pa-4 hrs sample. Several of the masses determined to be significant in table 2 are present in the Pa-4 hr, including *m/z* 5555.1, 1674.5 and 1792.8.

**Table 2:** The significant peaks with a p-value of less than 0.05 based on a Kruskal-Wallis test of the data.

Peak Number	m/z	p-value (Kruskal-Wallis)
41	1674.51	0.009
50	1792.82	0.009
101	2786.32	0.014
119	3155.06	0.014
174	5067.34	0.014
187	5555.09	0.014
197	6119.09	0.045
126	3418.68	0.037
14	1100.56	0.040
71	2208.52	0.049
231	8408.89	0.049

### Discriminatory Power of Biosignatures

**Figure 3:**
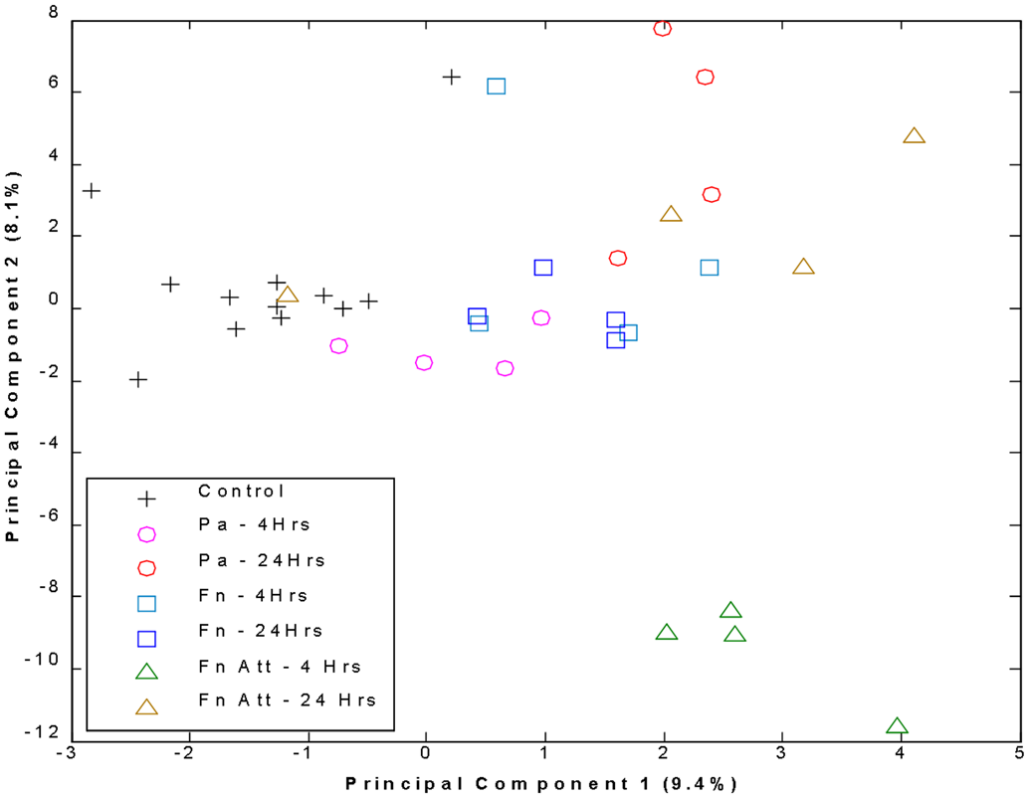
Distribution of replicate samples for seven classes of treatment: Control, PA-4 hrs, PA-24 hrs, Fn-4 hrs, Fn-24 hrs, Fn ATT-4 hrs, Fn ATT-24 hrs using two principle components.

As seen in Figure 3, some separation can be viewed for the different treatment classes from a standard Principal Component analysis (PCA). Missing data were imputed using a simple probability based method. If a peak was observed over 50% of the time the value was imputed as the average intensity, otherwise it was imputed as ½ of the minimum observed intensity. However, only ∼17.4% of the variability is explained in these first two components. Clearly the various treatments were distinct from the control samples of all time points. The Fn ATT samples also seemed to produce the most distinct response using these two principle components. The PCA gives some indication of distinctness of groups, however since imputation is not standard for MALDI, in-complete data methods, such as fingerprinting, are a more appropriate analytical method for MALDI.

Since our sample size was relatively small, most of the seven groups of interest, have only four samples, leave-one-out cross-validation (LOOCV) was used to classify each sample in a manner that was independent from the data used to build the reference fingerprints. Degree of association was used to build a predictive model on 35 of the 36 samples and a fingerprint was generated for the left out sample and probabilistically assigned to a class (Eq. 2). Table 3 summarizes the results with respect to each class in which 27 of the 36 samples are appropriately classified.

**Table 3:** The class predictions of the full 7 class Degree of Association model (Global Accuracy ∼75.0%).

		Predicted	Class					
		C	Pa-4	Pa-24	Fn-4	Fn-24	Fn-ATT-4	Fn-ATT-24
**True**	**C**	0.75	0.17				0.08	
**Class**	**Pa-4**		1.00					
	**Pa-24**			0.75				0.25
	**Fn-4**		0.25		0.75			
	**Fn-24**		0.25		0.25	0.25		0.25
	**Fn-ATT-4**						1.00	
	**Fn-ATT-24**			0.25				0.75

The statistical approach to classifying marker sets demonstrated an ability to discriminate classes of exposure 75% of the time. Not all treatment groups could be equally well defined using this approach. In the case of Pa-4 was accurately predicted for all four animals and Pa-24 three of the four times. The Fn-ATT-4, Fn-ATT-24 and Fn-4 were also predicted with relatively high confidence with all four mice for Fn-ATT-4 and three of four mice for the other two classes. The class that was most difficult to predict was Fn-24, where only one in four mice was correctly predicted and the other three mice predicted to be in three different treatment classes.

## Discussion

The goal of this study was to determine if the host response to aerosol exposure by different organisms would differ in the profile of peptides and small proteins captured in BALF. Three distinct types of organism challenges were used and compared to control animals. The samples were collected at two relatively short time points following exposure in an attempt to capture marker profile changes early in the host response. A number of different steps in the experimental process including aerosol exposure, sample purification and instrumental analysis could introduce bias in the data. Therefore, it was important to utilize effective experimental design for both exposure and analysis to limit the effects of the experimental process on data interpretation.

The differences in markers detected between the Fn and Pa exposures may have several possible explanations. Differences in reactivity between the major inflammatory component of the outer membrane, the LPS, between *F. novicida* and other gram negative organisms would indicate different host responses should be anticipated. The resulting pathology that is observed after *P. aeruginosa* and *F. novicida* infection is also distinct where the former induces an inflammatory response whereas the latter results in lung necrosis.

The differences between pathogens could also be seen using the total cell count. At four hours, only the Pa exposed animals appeared significantly different. However by 24 hours, the Pa and Fn treatments are distinct from the control and Fn-ATT treated animals. Finally, only the Fn treated animals had significant immune cell counts by 48 hours. While the cell count data differentiates each type of infection, this type of data may be difficult to acquire routinely in a clinical setting.

The use of MALDI-MS as a tool to profile and compare markers has several advantages. The ability to examine a large number of samples in a relatively small period of time with extensive analytical replication provides a distinct advantage over other methods for protein and peptide analysis. The tools for comparison of spectra data have been developed for several applications. However our approach to data analysis was developed through experience with bacterial identification and utilizes a different approach to spectral analysis than commercially available software packages. Our approach relies solely on peak presence/absence rather than peak intensity or area. This is intended to limit the affect of peak area variability that has been previously described [40], [41].

The results of this study indicate that the small protein and peptide markers analyzed by this method could be used as surrogates to indicate the type of exposure the mouse had received. Two types of respiratory pathogens and a non-pathogenic control were used in this study. For the earliest time point, four hours, the marker sets could be distinguished for all but one animal between each other and the control animals. While the markers are identified solely based on the measured mass, this study illustrates their potential utility. A second issue is that the biological origin of the markers is thought to be from the host, however that has not been determined experimentally. Isolation and identification of these markers will help elucidate their biological origin and significance.

One consideration is the change in marker sets between different time points, and presumably stages, of a given type of exposure and infection. In the case of *F. novicida*, the endpoint for all exposures is lethal. The change in marker sets from the initial exposure to a largely intracellular survival strategy may explain a corresponding shift in the marker sets observed in this study. A time-dependent shift in expression has been observed in previous work for some cytokines and chemokines following *F. novicida* exposure [37]. While those time-dependent changes were monitored over longer time intervals (24 to 48 hours), it may be likely that changes in expression also occur at earlier time points, such as those examined in this study.

Lastly, it is unknown if these markers are present in more accessible biological samples, such as breath condensate. However, previous studies examining different types of markers, such as lipid mediators, have demonstrated their presence in breath condensate [3]. A goal is to utilize these more accessible sample types in order to be useful for diagnosis of respiratory infection.

## Materials and Methods

### Materials

The acetic acid, triflouroacetic acid, acetonitrile, methanol, PMSF and ammonium hydroxide, ammonium acetate, ammonium bicarbonate was obtained from Sigma (St Louis, MO) at the highest purity available. The phosphate buffered saline (PBS) was obtained from Cell Gro (Voigt global inc, Lawrence, KS). Microcon molecular weight cutoff (MWCO) filters were purchased from Millipore (Burlington, MA). The 1 ml (100 mg bed volume) reverse phase C-18 T (P/N 8B-S004-EAK) and the 1 ml (30 mg bed volume P/N 8B-S029-TAK) strata X-C strong cation exchange solid phase extraction (SPE) cartridges were obtained from Phenomenex (Torrance, CA). The hydrophobic interaction (C-18) magnetic beads (MB-HIC) as well as protein and peptide mass calibration standards (P/N 219042) were purchased from Bruker Daltonics (Billerica MA).

### Bacterial Cultures

*Francisella novicida* U112 (Fn) and the *F. novicida* mglA mutant (Fn ATT) were provided by Dr. Francis Nano (University of Victoria, Canada). *Pseudomonas aeruginosa* PAK (Pa) was obtained from Dr. Steve Lory (Harvard University). Glycerol stocks were inoculated 1∶1000 into nutrient broth (TSB-0.1% L-cysteine for *F. novicida strains* and LB for *P. aeruginosa*) and incubated for 16 h at 37°C in a rotating platform incubator at 200 rpm. Bacteria were pelleted by centrifugation at 4°C, washed twice with PBS and resuspended in 5 ml cold PBS. Bacterial concentrations were estimated by optical density, using an OD450 of 0.2 as indicating a concentration of 2×10e9 CFU/ml (Fn and Fn ATT) or 2×10e8 CFU/ml (Pa). All bacteria were mixed thoroughly and diluted to 5×10^∧^9 CFU/ml in PBS in a total of 11 ml, of which 10 ml were used for each aerosolization.

### Aerosol exposure of mice

All animal experiments were approved by the Institutional Animal Care and Use Committee of the University of Washington (Seattle, Washington). Specific pathogen-free male and female C57BL/6 mice were obtained from Jackson Labs (Bar Harbor, ME) and were 8 weeks of age at the time of study. Mice were housed in filtered cages in a laminar flow rack (BioZone (Fort Mill, SC), and were permitted ad lib access to sterile food and water. For inhalation challenges, mice were placed in individual restraining tubes and exposed to aerosolized bacteria or PBS using a snout-only inhalation system (In-Tox Products, Moriarty, NM). Aerosols were generated using Mini-Heart Hi-flo nebulizers (Westmed, Tucson, AZ) driven at 44 psi with flow maintained at 2.5–2.6 L/min. Total airflow through the chamber was maintained at 17–26 L/min by negative pressure. Aerosol exposures were conducted for ten min, followed by 5 min purging with air. After each exposure the chamber was sterilized with Process NPD. Aerosol exposure experiments were conducted over 2 days using the following schedule:

Day 1:

1. PBS (8 mice)
2. Sterilization and clean
3. *F. novicida* (Fn - 6 mice)
4. Sterilization and clean
5. *F. novicida* mglA mutant (Fn-ATT - 6 mice)
6. Sterilization and clean

Day 2:

1. *P. auriginosa* (PAK) (Pa- 8 mice)
2. Sterilization and clean
3. *F. novicida* (Fn - 6 mice)
4. Sterilization and clean
5. *F. novicida* mglA mutant (Fn-ATT - 6 mice)
6. Sterilization and clean
7. PBS (8 mice)

Actual deposition of bacteria in the lungs was determined by quantitative cultures of lung tissues harvested from three sentinel mice immediately after each aerosol exposure, as described [42], [43]. The measured number of bacteria deposited lung with this treatment is provided in table 1.

### Collection of bronchial alveolar lavage fluid

Mice samples were harvested at T = 0 (PBS exposed only), 4H, 24H, and 48H (for PBS only); at 4H and 24H for *F. novicida, F. novicida* mglA mutant and *P. auriginosa* PAK post exposure. The samples were collected after the mice were anesthetized with pentobarbital and exsanguinated by heart puncture. The trachea and lungs were exposed and trachea cannulated with a catheter. Both lungs were lavaged by instilling 0.8 ml×4 of warm lavage fluid (0.9% saline with 0.6 mM EDTA). The lavage fluid was spun at 1200 rpm at room temperature and aliquoted with a final concentration of 1 mM PMSF.

### Culture supernatant preparation and acid extraction

A 0.2 mL portion of the bronchial alveolar lavage fluid (BALF) was applied to a Microcon 30 MWCO micro centrifuge spin filters. After centrifuging at 10,000 RCF for 30 minutes, the filtrate was collected. Acetic acid was added to a final concentration of 2% and the samples were allowed to incubate for 5 minutes at room temperature. The samples were then centrifuged again at 10,000×g for five minutes to remove any precipitated material.

The supernatants were applied to C-18 SPE cartridges placed on a vacuum apparatus that were previously conditioned according to the manufacturer recommendations. After washing with two 1 ml volumes of 0.1% TFA, the samples were eluted using 1 mL of 90% acetonitrile and 0.1% TFA into 1.5 mL eppendorf tubes. The eluted material was dried under nitrogen gas. Each sample was resuspended in 25 µL of 10% acetonitrile 0.1% TFA.

### Two stage SPE sample fractionation and cleanup

The mouse BALF samples were prepared for mass spectrometry in twelve sample batches using a twelve port manifold. As a result, a randomization order for preparation and analysis was employed. Table 2 contains the sample order used.

Two stages of SPE were employed in purification of the peptide fraction following a 30 kDa molecular weight cutoff purification step and acetic acid extraction. The strata-X-C column was conditioned with 2 mL of MeOH, 2 mL of 10 mM ammonium acetate (in 25% acetonitrile, 0.1% TFA pH 3.0), 2 mL of 500 mM ammonium acetate (in 25% acetonitrile, pH 6.8), 2 mL HPLC grade water, 2 mL 10 mM ammonium acetate (in 25% acetonitrile, 0.1% TFA pH 3.0) followed by vacuum to dry.

The 0.15 ml of acidified, filtered supernatant was added along with 0.85 mL of 10 mM ammonium acetate (in 25% acetonitrile, 0.1% TFA pH 3.0).After drawing the sample through the SPE bed, it was washed with 1 ml of 10 mM ammonium acetate (in 25% acetonitrile, 0.1% TFA pH 3.0), 1 ml of 100 mM ammonium acetate (in 25% acetonitrile, 0.1% TFA pH 3.0). Finally the sample was eluted with 1 mL of 80% MeOH/5% NH_4_OH. The eluate was dried under N_2_ to a volume of ∼50–100 µL and then brought up to a volume of 1 ml with 0.1% TFA.

Finally a C-18 hydrophobic interaction resin was used to clean-up marker for mass spectral analysis. The C-18 SPE was first conditioned using 2 mL of MeOH, 2 mL of H_2_O and 1 ml of 0.1% TFA. The reconstituted sample was then loaded and washed with 2 mL of 0.1% TFA. The peptides were then eluted into a clean 1.5 ml eppendorf tube with 1 ml of 90% Acn/0.1% TFA. The sample was dried under N_2_ flow and resuspended in 20 µL of 10% Acn/0.1% TFA or keep dry for longer term storage.

### Matrix assisted laser desorption/ionization mass spectrometry

The mass spectrometric analysis of purified proteins was performed using an Autoflex II MALDI tandem time of flight mass spectrometer equipped with a HIMAS ^tm^ detector (Bruker Daltonics, Billerica MA). Protein samples were spotted on a ground steel 384 multi target plate using 0.5 µL of sample followed immediately by 0.5 µL of matrix solution. Sinapinic acid (SA) matrix was used as a 10 mg/ml solution in 70% acetonitrile/0.03% TFA.

Five sample spots were deposited for each sample with successive samples in a statistically randomized order and deposited in a horizontal pattern. Five replicate spots for each sample type deposited in a vertical column. An external mass calibration was performed using protein standard mix II: Bovine myoglobin, cytochrome c, ubiquitin and insulin; spotted on adjacent locations.

Data was collected in linear mode from 1,000 to 30,000 *m/z* using a pulsed ion extraction delay time of 120 nsec. The resulting mass spectra consisted of 500 laser shots summed together. Automated data collection proceeded across each horizontal row before proceeding to the subsequent row containing the next set of replicate sample spots. This aided in randomizing data collection. Data was processed using available functions in the vendor software including a baseline subtraction and smoothing function (Savitzky-Golay with 0.2 m/z window) followed by peak picking using the centroid function and retained peaks with s/n greater than 5.

### Mass spectral data comparison

Mass spectra were compared using Clinprotools 2.0 (Bruker Daltonics, Billerica MA) for initial quality control of data. The goal of this analysis was to assess spectral quality and identification of missing data. Realignment and recalibration was done using the following settings within the software. Spectral preparation utilized the Convex Hull baseline function over a 1000–50,000 *m/z* range. Peak picking utilized a noise reduction threshold ignoring peaks below s/n two and picking peaks with s/n greater than five. The recalibration used a minimum resolution of 100 with a 0.2% maximum peak shift requiring 30% match to the calibrant peaks.

### Feature Extraction

Peaks are detected using an automated peak detection algorithm developed at PNNL [41] creating a list of peak locations (m/z) and associated intensity values from the mass spectrum of each sample. Targeting peptides, we consider only peaks having an m/z of less than 10,000. Following methods described in [41], the full set of spectra (all technical replicates from all experimental replicates) are aligned by combining the extracted peak lists and calculating an aligned peak location for each peak by averaging the m/z values from the samples in which that peak exists. This creates a peak table with each row representing a sample, each column an aligned peak, and each entry being either the intensity of a peak occurring in a sample or a missing peak flag if that peak doesn't occur in that sample. The code for the peak detection and alignment algorithms runs in MatLab® Version R2008a.

A Kruskal-Wallis test [44] was used to extract the relevant peaks from the full peak file using the MatLab® Version R2008a. The Kruskal-Wallis test is a nonparametric version of one-way analysis of variance, which tests the hypothesis that samples are drawn from the same population. The test will return a significant p-value if anyone of the defined groups has a normalized average peak area over the replicates that are significantly different from any of the rest. The Kruskal-Wallis test was based on seven groups in the data: (1) Control, (2) Pa (4 Hrs), (3) Pa (24 Hrs), (4) Fn (4 Hrs), (5) Fn (24 Hrs), (6) Fn-ATT (4 Hrs), and (7) Fn-ATT (24 Hrs). The results of the Kruskal-Wallis test are summarized in Table 2.

### Classification – Degree of Association

MALDI spectra were used to separate out the defined treatment and time groups. A mass spectral fingerprinting algorithm based on the degree of association between a MALDI reference library and the spectra of interest was used [40]. In particular the null hypothesis (*H_O_*) that a specific sample is from class *k* (e.g. Fn at 4 hours) is considered versus the alternative that the specific sample is not from class *k*. Assuming *H_O_* the sample under consideration can be described by the probability of observing peak *i* (*p_i_*). These probabilities are compared to the reference fingerprint of class *k* based on the set of fingerprint peaks that are differ and are in common between the sample fingerprint and the reference fingerprint – *Degree of Association* (DA):
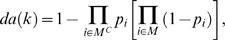
(1)


*M* is the fingerprint peaks that are not observed in the sample, i.e. missing, and *M^C^* is the complement, or observed peaks. All fingerprints are also subject to an occurrence filter that requires that a peak be observed in at least 60% of the replicates to be included in the fingerprint. The code to compute the DA for a fingerprint runs in MatLab® Version R2008a. The result of the model is the likelihood that that the sample is in the modeled class. Thus, the final classification of a sample *i* (*s_i_*) is taken to be the class *k* (*c_k_*) that the sample is classified in with maximum discrimination:
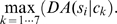
(2)


### Model Validation

A key step when fitting models that are to be used for prediction is to verify the results using cross-validation (CV). CV essentially is an approach to se gregate the data into independent sets so that the observations that are used to estimate the parameters of the model are not used to evaluate the classification accuracy [45]. In particular, with the DA fingerprint model leave-one-out CV (LOOCV) is used to evaluate the model accuracy. In LOOCV a single observation is left out of the model training phase. The trained DA fingerprint model is then run on this left out observation and the results are evaluated for classification accuracy. Iterating this process for each observation in the dataset returns a result for each observation that was attained in a manner that is independent from the data used to fit the model.
